# CpX Hunter web tool allows high-throughput identification of CpG, CpA, CpT, and CpC islands: A case study in *Drosophila* genome

**DOI:** 10.1016/j.jbc.2025.108537

**Published:** 2025-04-24

**Authors:** Martin Bartas, Michal Petrovič, Václav Brázda, Oldřich Trenz, Aleš Ďurčanský, Jiří Šťastný

**Affiliations:** 1 Department of Biology and Ecology, Faculty of Science, University of Ostrava, Ostrava, Czech Republic; 2 Department of Informatics, Mendel University in Brno, Brno, Czech Republic; 3 Department of Biophysical Chemistry and Molecular Oncology, Institute of Biophysics of the Czech Academy of Sciences, Brno, Czech Republic; 4 Faculty of Mechanical Engineering, Brno University of Technology, Brno, Czech Republic

**Keywords:** CpA islands, CpG islands, CpT islands, dinucleotide, *Drosophila*, genome analyses, web server

## Abstract

With continuous advances in DNA sequencing methods, accessibility to high-quality genomic information for all living organisms is ever-increasing. However, to interpret this information effectively and formulate hypotheses, users often require higher level programming skills. Therefore, the generation of web-based tools is becoming increasingly popular. CpG island regions in genomes are often found in gene promoters and are prone to DNA methylation, with their methylation status determining if a gene is expressed. Notably, understanding the biological impact of CpX modifications on genomic regulation is becoming increasingly important as these modifications have been associated with diseases such as cancer and neurodegeneration. However, there is currently no easy-to-use, scalable tool to detect and quantify CpX islands in full genomes. We have developed a Java-based web server for CpX island analyses that benefits from the DNA Analyzer Web server environment and overcomes several limitations. For a pilot demonstration study, we selected a well-described model organism *Drosophila melanogaster*. Subsequent analysis of the obtained CpX islands revealed several interesting and previously undescribed phenomena. One of them is the fact, that nearly half of long CpG islands were located on chromosome X, and that long CpA and CpT islands were significantly overrepresented at the subcentromeric regions of autosomes (chr2 and chr3) and also on chromosome Y. Wide genome overlays of predicted CpX islands revealed their co-occurrence with various (epi)genomics features comprising cytosine methylations, accessible chromatin, transposable elements, or binding of transcription factors and other proteins. CpX Hunter is freely available as a web tool at: https://bioinformatics.ibp.cz/#/analyse/cpg.

CpG islands are regions within nucleic acids characterized by a high frequency of CpG dinucleotides, which are prone to methylation (1). The likelihood of CpG methylation is influenced by several factors, including the genomic context, the presence of specific DNA-binding proteins, and the activity of DNA methyltransferases (2, 3). For example, CpG islands located in gene promoters are typically unmethylated in normal cells, allowing active transcription, whereas methylation of these regions is often associated with transcriptional silencing (4). This dynamic regulation is important for processes such as tissue-specific gene expression and cellular differentiation. Notably, genes such as *MLH1* and *BRCA1* are regulated through CpG island methylation, with aberrant methylation patterns linked to cancer development (5, 6). Cytosine methylation is an enzymatically driven process catalyzed by DNA methyltransferases, such as DNMT1, DNMT3A, and DNMT3B (in mammals), which add a methyl group to the C5 position of cytosine (7). Beyond these core enzymes, other proteins, including methyl-CpG-binding domain proteins (MBDs) and transcription factors, play critical roles in interpreting and modulating methylation marks (8). These proteins contribute to the recruitment of chromatin remodelers and histone modifiers, further influencing gene expression (9, 10).

Since the systematic documentation of CpG islands in the 1980s (11, 12), substantial evidence has emerged highlighting their roles in development (13), cancer (14), and aging (15). For instance, CpG island methylation is essential for human X-chromosome inactivation during development and the establishment of genomic imprinting (16). In cancer, hypermethylation of CpG islands in tumor suppressor gene promoters, such as p16 and RB1, leads to their silencing and contributes to tumorigenesis (17). Similarly, age-related changes in CpG island methylation have been implicated in the dysregulation of genes associated with cellular senescence and age-related diseases (18). While methylated cytosines are predominantly found in CpG islands, non-CpG methylation (*e.g.*, CpA, CpT, and CpC) has also been identified, particularly in embryonic stem cells and neurons (19, 20). CpX islands, defined here as cytosine followed by any nucleotide (G, A, T, or C), have garnered increasing interest due to their emerging roles in cancer (21) and brain function (22). For example, non-CpG methylation has been shown to regulate neuronal activity and synaptic plasticity, while aberrant methylation patterns at CpX sites have been linked to cancers (21). These findings underscore the need to explore CpX islands further to uncover their biological significance.

The motivation to identify CpX islands is twofold. First, these regions can be methylated and regulate molecular processes such as transcriptional repression, chromatin remodeling, and genomic stability. Second, CpX islands can promote the formation of non-canonical DNA structures, such as left-handed Z-DNA, which is associated with CG/GC and CA/GT dinucleotides (23). Z-DNA formation has been implicated in transcriptional regulation and genomic instability, suggesting that CpX islands could mark regions of functional and structural importance. *Drosophila melanogaster*, commonly known as the fruit fly, is a widely used model organism in biological research due to its well-characterized genome and rapid life cycle. Despite decades of study, the functional importance of CpG islands in *Drosophila* remains unclear (24), as its genome exhibits minimal CpG methylation (25). Some studies even suggest that the *Drosophila* genome lacks canonical CpG islands altogether (26). However, recent research has highlighted the potential roles of non-CpG methylation in *Drosophila* development and gene regulation, making it a promising model for studying epigenetic mechanisms (27). Investigating CpX islands in *Drosophila* could provide new insights into fundamental molecular processes and their evolutionary conservation.

Here, we present CpX Hunter, a user-friendly and freely accessible web-based tool designed for whole-genome investigation of cytosine-based dinucleotide islands, including both canonical CpG dinucleotides and their adenine, thymine, or cytosine variants. Our tool builds upon the algorithm formulated by Takai and Jones (28), offering enhanced functionality such as customizable search parameters and the ability to analyze non-CpG dinucleotides. In this study, we applied CpX Hunter to the *D. melanogaster* genome, conducting a pilot analysis to identify CpX islands and their overlap with various (epi)genomic features, including ChIP-seq, Bisulfite-seq, ATAC-seq, and DNAse-seq data. By enabling the systematic identification and characterization of CpX islands, our tool provides a valuable resource for advancing the understanding of epigenetic regulation and its implications in development, disease, and evolution.

## Results

### Development of CpX Hunter and benchmarking

**Figure 1:**
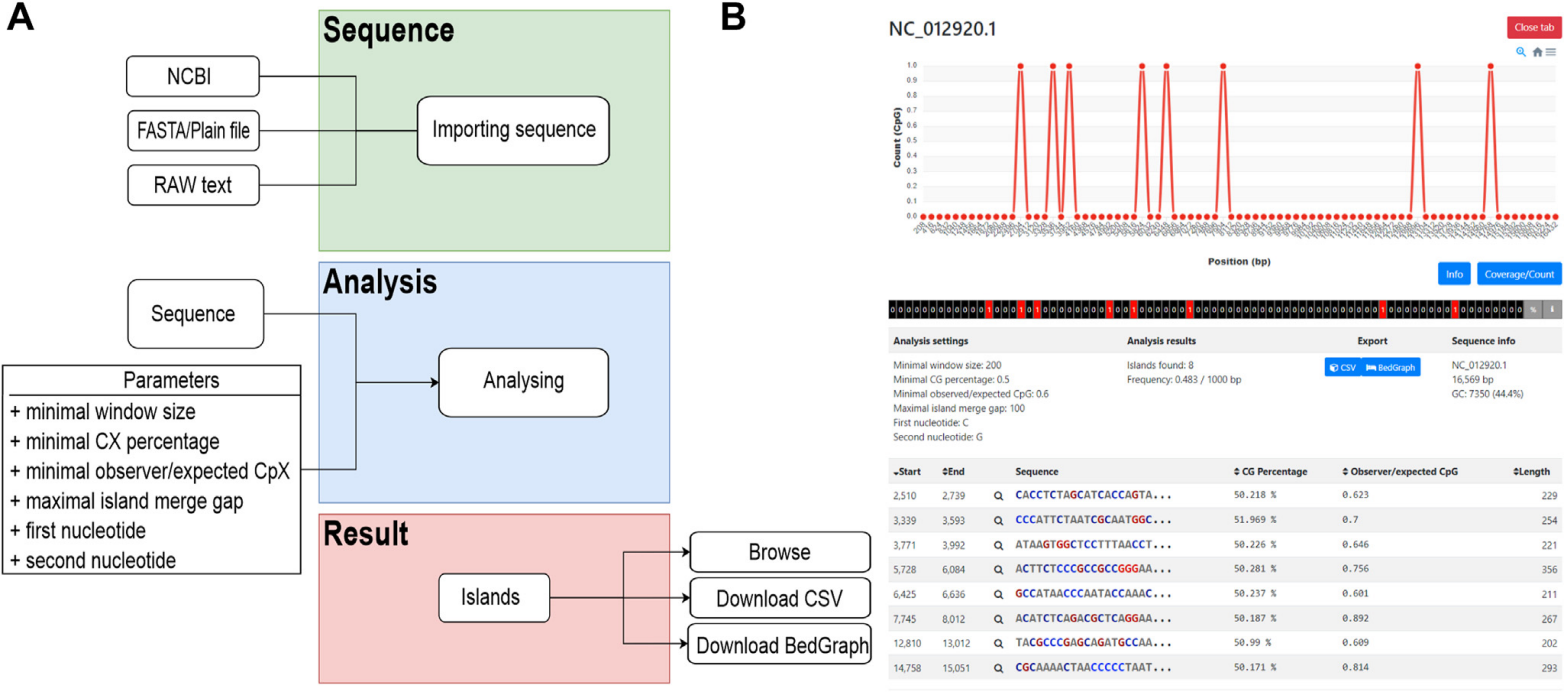
**CpX Hunter workflow and an example of a CpX Hunter result.***A*, CpX Hunter workflow consists of three main parts: sequence import (*green*), analysis (*blue*), and results (*pink*). *B*, visualization of results in the web browser window from upper part: sequence ID, histogram of CpX locations in the tested sequence, number of CpX in locations, analysis settings, sequence statistics, and individual results.

The overall scheme of the CpX Hunter workflow is depicted in Figure 1*A*. CpX Hunter uses an interactive web interface with Asynchronous JavaScript and XML (AJAX) to dynamically update its display of analysis results (Fig. 1*B*). Along with comprehensive statistics and sequence characteristics, it displays a heatmap of the CpX island distribution and allows multiple simultaneous analyses, each in its own tab. The results can be exported in BedGraph and .csv formats for further study or record keeping. Information about each identified CpX island includes the following:

#### Island start and end positions

Precise genomic location information can be obtained using genomic coordinates.

#### Sequence

Additional analysis can be performed on the expandable nucleotide sequence of the CpX island.

#### CX percentage

Reflects the density of cytosine-based dinucleotides within the island.

#### Observed to expected CpX ratio

Indicates the regulatory significance of the island.

#### Island length

Provides information about the sequence length of the CpX island.

#### Export options

The tool allows for the export of results in formats compatible with the genome browser for visual analysis and the overlay of CpX island data on genomic maps. The results can be downloaded in the .csv format with many spreadsheet programs and in the BedGraph format compatible with tools for genome annotation, including UCSC Genome Browser (29). Both export formats contain parameters including start and end position, shortened sequence window, GC percentage, observer to expected CpX ratio, and length of the window.

#### Use of the API and export options

The capabilities of CpX Hunter are expanded by the DNA Analyser API, which allows integration with unique scripts or web services for more extensive bioinformatics analyses and automated workflows.

### Benchmark

To test our new Java-based CpX Hunter tool, we performed several tests and made comparisons with the original CpG Python-based code. Both tools identified the same number of CpG islands, but our new algorithm was significantly faster when tested using the same hardware (server computer: Intel Xeon Gold 6230, 80 cores, RAM: 92 GB). We tested the human mitochondrial DNA sequence (NCBI ID: NC_012920.1, 16,569 bp), human chromosome 22 (NCBI ID: NC_060946.1, 51,324,926 bp), and human chromosome 1 (NCBI ID: NC_060925.1, 248,387,328 bp). The mitochondrial DNA was analyzed around 3.6 times faster using our algorithm (0.017 s *versus* 0.062 s for our code and the Python code, respectively). The advantage of our new implementation was evident, especially for the longer full chromosome sequences. Human chromosome 22 was analyzed in 2.063 s using our code, whilst this took 175.745 s with the Python code. Moreover, the far larger human chromosome 1 could be analyzed within 8.333 s using our code, whilst this took 889.505 s with the Python code. Thus, the advantage of the new algorithm implementation is marked, especially for long sequences, where the speed is ˃80 times faster (for chromosome 22) and ˃100 times faster (chromosome 1) compared to the previous implementation. In addition to the fully scalable features, the ability to analyze not only CpG islands but also all other dinucleotide possibilities, within seconds, brings new possibilities for effective genome analyses.

### CpX Hunter revealed interesting patterns of CpG, CpA, and CpT islands distribution in the *D. melanogaster* genome

**Figure 2:**
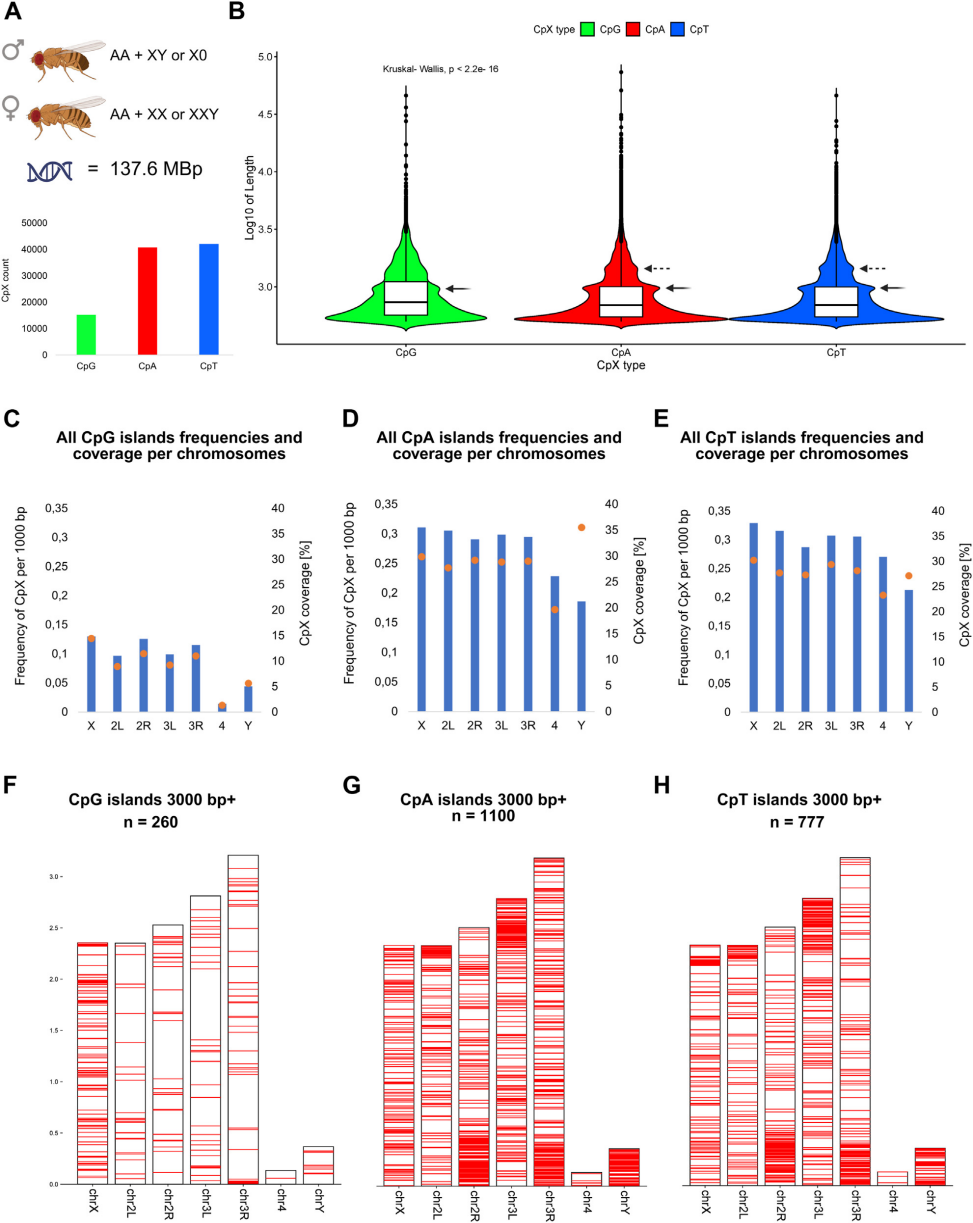
**Analysis of CpX islands occurrence and distribution in *Drosophila melanogaster* genome.***A*, basic chromosomal karyotype of *Drosophila melanogaster* and total counts for particular CpX island types. *B*, Violin plots (with additional boxplots) to represent a distribution of CpX lengths on a log10 scale. *C*–*E*, combined plots representing CpX frequencies per 1000 bp (*blue* bars) and CpX coverage (*orange* dots) per particular chromosomes. (*F*–*H*) Chromosomal plots showing the distribution of CpX islands longer than 3000 bp (each start position is visualized as a thin horizontal *red* line).

Here, we present the first complex analysis of CpX occurrence and distribution in the genome of model organism *D. melanogaster*. As this genome has only 5 autosomal contigs (2L, 2R, 3L, 3R, 4), 2 gonosomes (X and Y), and mitochondrion, it is an ideal candidate for concise visualization (Fig. 2). Using the reference version of the *D. melanogaster* genome (dm6), we were able to identify 15,180 CpG islands, 40,704 CpA islands, and 42,045 CpT islands (Fig. 2*A*), original .bed files are enclosed (Supplementary material 1, *A*–*C*). No CpC island was identified. The median length of CpG islands was 733 bp, and both CpA and CpT islands had significantly shorter median lengths of 692 bp and 694 bp, respectively. The distribution of CpX islands' length is depicted in Figure 2*B* on the logarithmic scale. According to expectations, the most frequently represented lengths were close to the CpX Hunter threshold of 500 bp. Interestingly, for both CpX island types (mostly for CpT and CpA islands), there was a second peak of occurrence around the island length of 1000 bp (black arrows). For CpT and CpA islands, even a third smaller peak occurred around 1500 bp (dashed arrows). Regarding frequencies of CpX islands, it is clearly visible (Fig. 2, *C*–*E*) that CpG islands are minimally 2.5 times “rarer” than CpA and CpT islands, considering particular chromosomes. The most distinct difference in frequencies was for chromosome 4, where CpG islands had a frequency of 0.014 per 1000 bp and CpT islands had a frequency of 0.270 per 1000 bp, *i.e.*, nearly 20 times higher (Table 1). A similar trend was observed for CpX coverage, where CpG islands covered a maximum of 14.5% in the case of chromosome X, but CpT islands covered 30.3% and CpA islands 29.9% (for the same chromosome) (Table 1). An interesting phenomenon was observed in the case of CpA islands coverage on chromosome Y which has the lowest CpA islands frequency value (0.186) from all chromosomes but the highest coverage (35.5%), indicating that CpA islands on chromosome Y are longer than CpA islands on the rest of chromosomes (Table 1). That was subsequently statistically tested and indeed, CpA islands found on chromosome Y are significantly longer than those found on the rest of the chromosomes (*p*-value = 1.8e-14, non-parameter Wilcoxon test for two group comparison was used), the median length of CpA islands on chromosome Y was 844 bp, whereas the rest of chromosomes had a median value of 690 bp.

**Table 1:** Basic characteristics of CpG, CpA, and CpT islands in *Drosophila melanogaster*

Islands type	Characteristics	X	2L	2R	3L	3R	4	Y	MT
	chr length [bp]	23,542,271	23,513,712	25,286,936	28,110,227	32,079,331	1,348,131	3,667,352	19,524
	GC [%]	42.5	42	42.5	41.5	42.5	35	39.5	18
CpG islands	count CpG	3066	2269	3179	2784	3701	19	162	0
	sum of CpG [bp]	3,407,151	2,105,771	2,906,926	2,589,377	3,542,220	17,907	206,793	0
	median length CpG [bp]	794,5	722	718	718	711	687	903	0
	freq CpG per 1000 bp	0.130	0.096	0.126	0.099	0.115	0.014	0.044	0
	coverage CpG [%]	14.5	9.0	11.5	9.2	11.0	1.3	5.6	0
CpA islands	count CpA	7316	7190	7358	8391	9457	308	681	3
	sum of CpA [bp]	7,028,647	6,520,015	7,384,236	8,115,587	9,315,582	265,069	1,302,828	1962
	median length CpA [bp]	698	678	687	700	694	629	844	557
	freq CpA per 1000 bp	0.311	0.306	0.291	0.299	0.295	0.228	0.186	0.154
	coverage CpA [%]	29.9	27.7	29.2	28.9	29.0	19.7	35.5	10.0
CpT islands	count CpT	7751	7420	7270	8637	9814	365	781	7
	sum of CpT [bp]	7,121,642	6,516,279	6,904,591	8,265,926	9,023,611	313,929	995,309	5765
	median length CpT [bp]	699	676	698	702	689	666	807	517
	freq CpT per 1000 bp	0.329	0.316	0.288	0.307	0.306	0.271	0.213	0.359
	coverage CpT [%]	30.3	27.7	27.3	29.4	28.1	23.3	27.1	29.5

In columns, there are particular *Drosophila* chromosomes together with the mitochondrial genome (MT) and rows contain general characteristics (chromosome length and GC content) and then specific characteristics regarding CpG islands, CpA islands, and CpT islands.

Finally, we have focused on the genomic distribution of the longest CpX islands with a threshold of 3000 bp and longer. There were 260 such CpG islands (covering 0.88% of the genome), 1100 CpA islands (covering 4.35% of the genome), and 777 CpT islands (covering 2.65% of the genome). Regarding long CpG islands, the interesting fact is that nearly half of these sites (113 out of 260) were found on chromosome X. Chromosomal distribution of long CpA and CpT islands revealed another fascinating phenomenon, as there were enriched clusters of islands at the sub centromeric ends of chromosomes 2L and 3L (5′-prime), and at the sub centromeric ends of chromosomes 3L and 3R (3′-prime). *There is an important thing to realize: 2L and 2R, together with 3L and 3R chromosomes in Drosophila are in fact left (L) and right (R) arms of chromosomes 2 and 3* (30). In addition, chromosome Y was overall enriched in long CpA and CpT islands.

### Analysis of CpX overlays with various (epi)genomic features revealed interesting functional consequences

To find out whether some significant overlaps of CpX islands and functional (epi)genomics signatures exist, we employed the ChIP-Atlas database, and its feature called Enrichment Analysis. The table below summarizes what type of analyses were done (Table 2). We have focused on potentially biologically relevant significant enrichments (or depletions), as sometimes the “*p*-values” seemed to be very low (*i.e.*, statistically significant), however, real “effect size” expressed as fold ratio was negligible. Therefore, we chose the following criteria to arbitrarily judge potential “biological significance”: Log *p*-value < −10, fold enrichment at least 1.5 (for depletion 0.5), and support from at least 10 ChIP-Atlas samples.

**Table 2:** Overview of performed enrichment analyses with ChIP-Atlas datasets

Experiment type	CpG	CpA	CpT	CpG 3000+	CpA 3000+	CpT 3000+
ATAC-seq (Accessible chromatin)	↑	∼	∼	∼	↓	↓
Bisulfite-seq (Cytosine methylations)	↑	∼	∼	∼	∼	∼
DNAse-seq (Accessible chromatin)	∼	∼	∼	∼	↓	↓
Histone modifications	↑↓	↓	↓	↑	↑↓	↑↓
RNA Pol II	↑	↓	∼	∼	↓	↓
Transcription factors and others	↑↓	↓	↓	∼	↑↓	↓

The upward arrows indicate biologically significant enrichment and the downward arrows show biologically significant depletion of particular (epi)genomics features within particular CpX islands and their long (3000 bp+) subsets.

ATAC-seq datasets group experiments dealing with chromatin accessibility across the *Drosophila* genome. The most significant overlays were found in the case of CpG islands, where mainly *enrichments* were observed, *i.e.*, CpG islands were sites where accessible chromatin was presented up to 3.5 times more frequently than in the control (100 permutated dataset of CpG islands). In contrast, there were significant *depletions* of accessible chromatin within long (3000 bp+) CpA and CpT islands, having up to 5 times less accessible chromatin than expected (Supplementary Material 2*A*). A similar phenomenon was also observed in the case of CpX islands overlays with DNAse-seq experiments - long CpA and CpT islands showed significant depletion of “open” chromatin (Supplementary Material 2*C*).

According to expectations, overlays of CpX islands with bisulfite sequencing experiments revealed that hypermethylated regions were significantly enriched (up to 4 times) within CpG islands. For CpA and CpT islands, only mild enrichments or depletions were observed in some experiments (Supplementary Material 2*B*).

Histone modifications, comprising mainly several types of methylations and acetylations, are quite various groups of features, where, *e.g*., in the case of CpG islands, both significant enrichments and depletions were observed (depending on cell types, developmental stage, and types of particular histone antigens). (Supplementary Material 2*D*). The highest enrichment in CpG islands was observed in the case of H3 acetylation in adult ovary cells (6.08). Interestingly, there were also several statistically significant total depletions of histone modifications within CpG islands, *e.g.*, for H3K36 di-methylation (H3K36me2). Interestingly, within CpA islands in general, the histone modifications were significantly depleted, whereas, within the long subset of CpA islands, there were both significant enrichments (mainly of H3K9 methylations, up to 3.75 times) and depletions. Considering CpT islands, they were also generally depleted in histone modifications, and within long CpT islands, there were both significant depletions and enrichments.

Overlays of CpX with RNA polymerase II chromatin interaction sites revealed significant enrichments mainly within CpG islands. CpA islands, together with their long subsets, were significantly depleted in RNA polymerase II interactions. For CpT islands, only their long subset was significantly depleted in RNA polymerase II interactions (Supplementary Material 2*E*).

**Figure 3:**
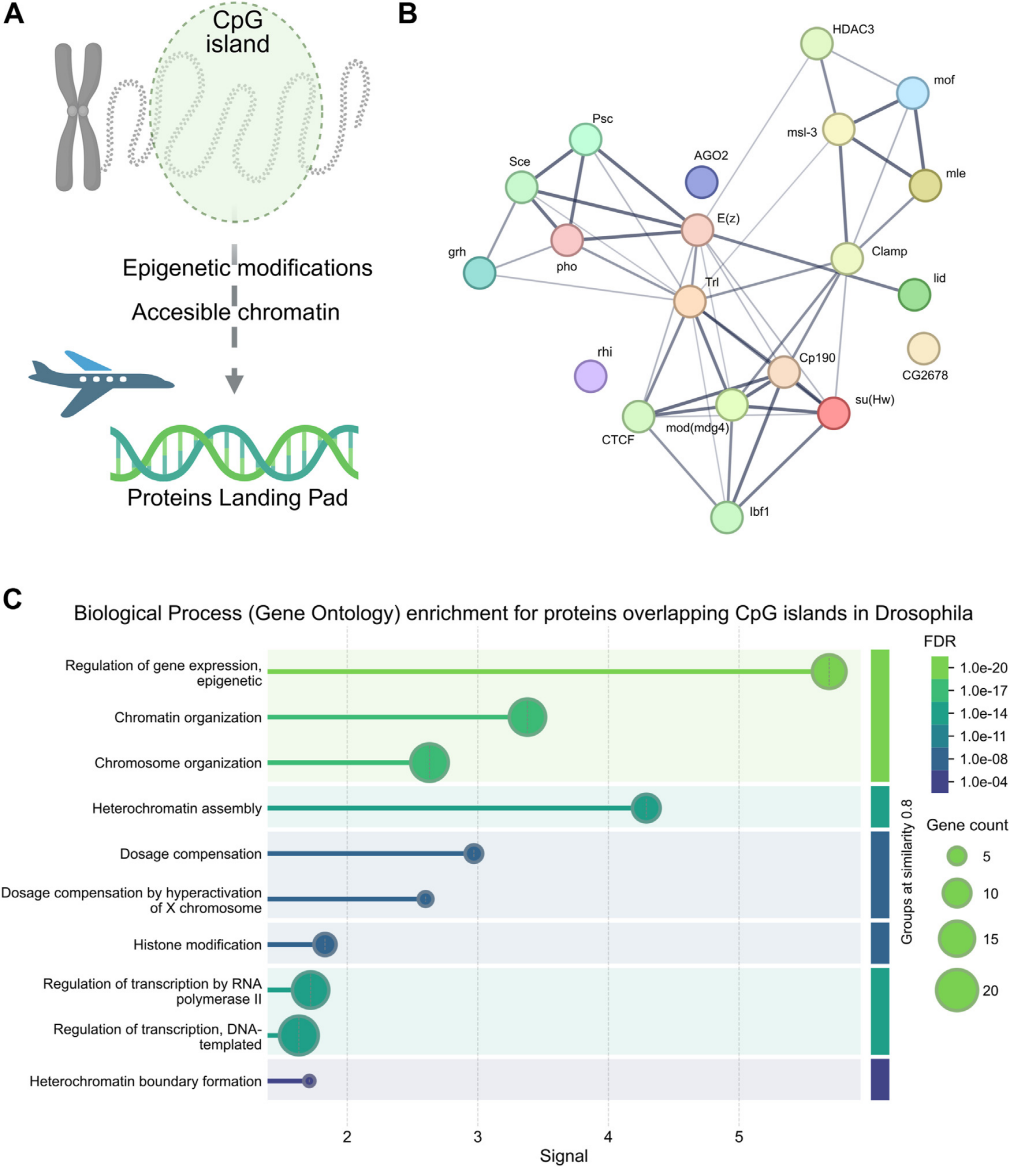
**Proteins significantly enriched within CpG islands in Drosophila.***A*, a simple scheme of a single representative CpG island within chromatin that, thanks to epigenetic modifications and local chromatin accessibility, forms something like a “landing pad” for many proteins. *B*, a STRING functional interaction network was constructed from the 20 most enriched proteins within CpG islands. The thickness of lines indicates the confidence of functional interconnections. *C*, graphical representation of most enriched GO Biological processes together with false discovery rates (FDR).

Finally, many transcription factors were significantly enriched in the regions of the CpX islands (Supplementary Material 2*F*). Considering previous findings (significant enrichments of epigenetic modifications and accessible chromatin within CpG islands), this is not so surprising and rather offers a logical explanation of the observed phenomenon (Fig. 3*A*). The most statistically enriched proteins (with Log P-val ≤ −100) were further used for constructing a functional interaction network (Fig. 3*B*). This revealed that most of them are strongly, functionally interconnected, and a substantial part of them even physically interact with each other. Statistical analysis of enriched biological processes further revealed that the most enriched gene ontology (GO) terms were related to chromatin organization and epigenetic regulations of gene expression (Fig. 3*C*). Regarding CpA and CpT islands, they showed mostly significant depletions for many proteins, indicating these sites are not generally preferred for DNA–protein interactions.

**Figure 4:**
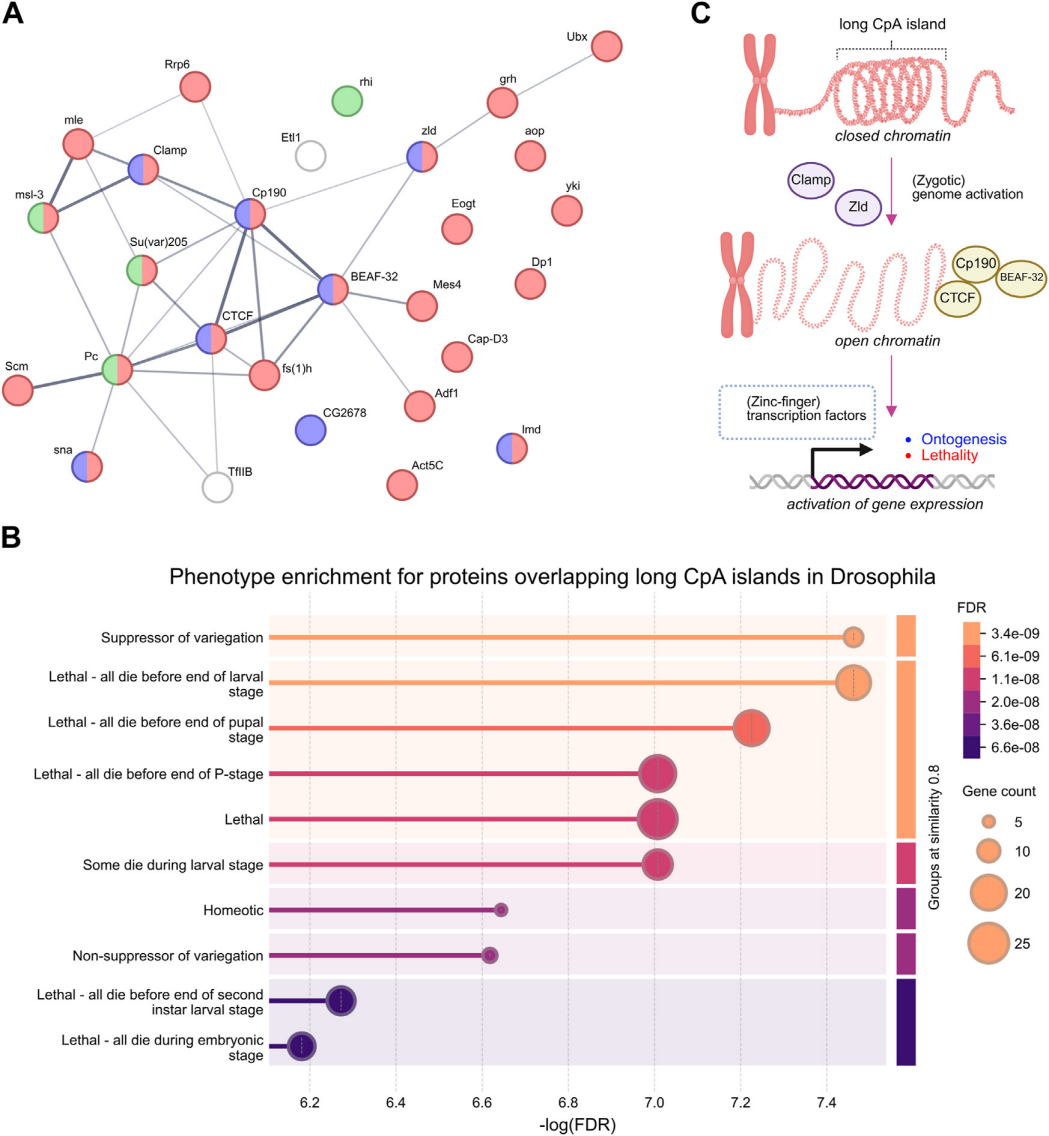
**Proteins significantly enriched within long CpA islands in Drosophila.***A*, a STRING functional interaction network is constructed from 28 of the most enriched proteins within long CpA islands. The thickness of lines indicates the confidence of functional interconnections. Proteins involved in the “Lethal” *Drosophila* Phenotype (Monarch ID: FBcv:0000351) are highlighted in *red*. The *blue* color is for proteins containing the Zinc finger C2H2 superfamily protein domain. Proteins containing chromo-like domains are in *green*. *B*, graphical representation of most statistically enriched *Drosophila* Phenotypes (Monarch). *C*, hypothetical function of long CpA islands in *Drosophila* – long CpA islands are mostly in the state of closed chromatin shaped by m6A epigenetic marks, but during certain molecular events, they attract specific proteins in order to loosen chromatin and allow transcription of target genes finally leading to the next ontogenesis stage or cellular/tissue death.

Unexpectedly, several transcription factors seem to prefer long CpA islands. To the best of our knowledge, there is no mention of any proteins preferring such (or similar) sites in the literature so far. We identified a total of 28 such proteins (Log P-val < −10) that (at least under specific conditions depending on experimental designs of particular ChIP-Atlas-deposited experiments) prefer long CpA islands. Constructed functional interaction network revealed their tight connection (Fig. 4*A*) and some of them are even physically interacting - this is the case for mle and msl-3 proteins, for Scm and Pc proteins, and for BEAF-32, Cp190, and CTCF proteins. Interestingly, most of these proteins are related to lethal *Drosophila* phenotypes (Fig. 4*B*), which led us to the hypothesis that CpA islands could serve as chromosomal death-promoting regions (in the meaning of subsequent cellular fate), and/or as important regulator sites during embryogenesis. Regarding their genomic localization, long CpA islands are predominantly found in subcentromeric regions of autosomes 2 and 3, and within the whole Y chromosome, but are more or less dispersed also on the rest of all chromosomes (Fig. 2*G*).

To inspect the near surroundings of particular long CpX islands, also so-called CpX shores (which are standardly defined as sequences 2 kbp upstream and 2 kbp downstream of CpG islands (31)) were acquired and used as input datasets for overlays with the above-described (epi)genomic features. The resulting data are enclosed in Supplementary Material 2, *A*–*F* on separate lists. The observed enrichments or depletions (if any) for CpX shores are generally less significant when compared to CpX islands, indicating that the role of CpX shores in *Drosophila* is rather minor.

Finally, we decided to put the obtained data in the context of transposable elements (TEs), so we downloaded the bedgraph coordinates (Supplementary material 3) of all natural transposable elements (TE) in the *D. melanogaster* genome from the Flybase database (32). In total, there were 5889 TEs in 153 categories. The most abundant TE was INE-1 (with 2235 counts within the *Drosophila* genome) and it overlapped with CpG islands only in 1% of cases, whereas with CpA and CpT islands in 25% and 35% of cases (such overlap is insignificant with respect to CpA and CpT islands genome coverage close to 30%). To identify TE types enriched within particular CpX islands, we used an arbitrary threshold of 75% (of TEs that must overlap with CpX islands) and a minimal total amount of TEs to be at least 10 (to avoid random overlaps). While several TE types overlap with CpX rarely, others overlap with various CpX often, and sometimes one particular TE locus overlaps even with more than one CpX island (resulting in percentual values over 100). Complete results of *Drosophila* TEs and CpX islands overlap is enclosed in Supplementary material 4. The best overlaps are shown in Table 3 below.

**Table 3:** Top eight most enriched TEs within particular CpX islands

CpG islands			CpA islands			CpT islands		
Rt1a (30)	48	160%	roo (144)	388	269%	Transpac (12)	27	225%
G6 (15)	21	140%	gypsy2 (16)	36	225%	Stalker2 (17)	37	218%
Rt1b (60)	63	105%	opus (44)	68	155%	opus (44)	88	200%
opus (44)	40	91%	springer (22)	34	155%	Tirant (25)	45	180%
Max (24)	21	88%	flea (29)	43	148%	17.6 (29)	50	172%
R1A1 (31)	25	81%	Max (24)	32	133%	HMS-Beagle (24)	41	171%
GATE (19)	15	79%	gypsy3 (15)	20	133%	gypsy5 (10)	17	170%
gypsy8 (56)	42	75%	GATE (19)	25	132%	297 (89)	146	164%

The total counts of particular TEs are in brackets. In the other two columns, the overlap counts of particular TE types and the total percentual overlaps with CpX islands are always indicated.

## Discussion

In this article, we have presented a new web-based, scalable, and user-friendly tool for the prediction of CpX islands occurrence. We demonstrated that this tool is capable of processing even whole eukaryotic chromosomes in an extremely short time and provided an exemplary analysis of the *D. melanogaster* genome as a model eukaryotic organism with a low number of chromosomes suitable for good visualization. In addition, several interesting observations were made—one of them was the overrepresentation of long CpG islands on chromosome X, and we initially thought that it could be related to a known phenomenon of X-inactivation driven by CpG methylation (33, 34). *Drosophila* females have two X chromosomes (like humans), and molecular mechanisms to secure dosage compensation must exist. However, *Drosophila*, according to the literature, uses a twofold increase in X-linked gene expression in males, rather than X-inactivation in females (27, 35). Long CpG islands located predominantly on *Drosophila* X chromosomes could therefore have still undescribed role(s).

One of the most unexpected findings was the significant enrichment of several proteins within long CpA islands. In addition, these proteins formed a strong interaction network with many statistically overrepresented terms. The mechanism(s) by which these proteins prefer/recognize long CpA genomic regions is unclear and will require additional effort and wet lab experiments. Protein Cp190, which was enriched more than sixfold in long CpA islands, is considered to serve as a cofactor of architectural proteins (36). Also, this protein, together with CTCF, BEAF-32, Clamp, Zelda (zld), sna, lmd, and CG2678, contains C2H2-type zinc finger domains. The function of *Drosophila* CTCF seems to be quite different from vertebrate CTCF (where it plays a crucial role in chromosomal loop formation) (37). Additionally, it was found that *Drosophila* CTCF, Cp190, and BEAF-32 proteins are not required to generate topologically associating domains (TADs) (38). Proteins Clamp and zld are considered to play an important role in *Drosophila* zygotic genome activation by modulating chromatin accessibility (39). Long CpA islands were significantly depleted in the amount of accessible chromatin; therefore, proteins Clamp and zld could drive the expression of genes located within or in close proximity of long CpA islands by increasing chromatin accessibility of such loci (Fig. 4*C*). Another interesting protein, Polycomb group protein Pc, is known N6-methyladenosine (m6A) binder (40). This fact could indirectly indicate that long CpA islands contain a substantial amount of m6A epigenetic marks. The only protein significantly enriched in long CpT islands was BEAF-32 (enriched more than 4 times in long CpT islands). BEAF-32 protein was shown to physically interact with the polybromo subunit of PBAP, a SWI/SNF-class chromatin remodeling complex (41). Otherwise, long CpT islands were depleted in protein binding sites acquired from accessible ChIP-seq experiments.

Obviously, the molecular functions of CpA and CpT islands are still to be determined. In this article, we aimed for the first attempt leading to their definitions (which may and most probably will evolve during the course of time) using the model organism *D. melanogaster*. We expect that the application of the same protocol will lead to surprising discoveries also in other animal genomes, including humans. We expect that the situation in plants and other non-animal species may be even more complex due to overall differences in their epigenomics landscape (42).

Interestingly, no single CpC island was found in the whole genome of *Drosophila*. This may be the result of a too strict threshold, which was originally developed for the detection of CpG islands. Although the same threshold worked well for CpA and CpT islands detection, the situation with CpC islands seems to be a little bit complicated, as generally, organisms tend rather avoiding long homopurine or homopyrimidine nucleotide tracts that can lead to genomic instability. In addition, C-rich sequences are in fact G-rich sequences on the opposite strand, and resulting G-quadruplex structures arising from such long G-rich regions (minimum threshold length for CpX islands was 500 bp) would form significant obstacles for DNA replication (43), and transcription (44). Finally, we cannot exclude the possibility that once the parameters of the CpC search are manually loosened, many (potentially biologically relevant) CpC islands will be detected.

Overlaps between CpX islands and known TEs in *Drosophila* revealed several interesting phenomena. At first, several TE types showed high overlaps with particular CpX islands (*e.g.* TE “297” was exclusively enriched within CpT islands, gypsy3 within CpA islands, and gypsy8 within CpG islands). Secondly, some TEs were spanned by more than one CpX island, which is most probably a consequence of TE length (whereas the minimal length of CpX was set up to be 500 nucleotides, some TEs in *Drosophila* are much longer and therefore can accommodate several (even various) CpX islands. TEs in *Drosophila* can be inserted near or within regulatory regions (45), including promoters (46), and hypothetically act as cis-regulatory elements, thereby modulating gene expression by introducing new transcription factor binding sites or altering chromatin accessibility and structure. The co-occurrence of some types of TEs and CpX islands should be investigated further to reveal its mechanistic basics.

CpX Hunter provides a user-friendly interface for analyzing dinucleotide repeats and has significant potential to contribute valuable insights into the structural dynamics and biological functions of these unique DNA conformations. We expect that this tool will help a wide range of researchers generate many new hypotheses and will facilitate exciting discoveries within the field.

## Experimental procedures

### CpX Hunter development and integration

CpX Hunter offers a complete suite for DNA sequence analysis and is incorporated into the DNA Analyzer Web server, which integrates several tools such as G4Hunter (47) and Palindrome Analyzer (48). CpX Hunter is based on the algorithm formulated by Takai and Jones (28) and thanks to the server-based Java implementation, is able to identify dinucleotide islands (CpX) within whole genomes. It features a high performance back-end and a user-friendly web interface for easy analysis and interactive visualization of results. All imported sequences and analyses are stored in a database for data persistence and future retrieval purposes. An application programming interface (API) is available in the web application to integrate with a wide set of sequence analysis tools and help facilitate batch processing.

### Procedure for input and analysis

Users can upload files directly in FASTA or plain text format, use NCBI IDs to upload individually, or upload DNA sequences in bulk directly from the NCBI Genome database. Additionally, the web application allows direct clipboard input for rapid sequence testing. All uploaded sequences can be tagged for easy organization. Sequences up to 2048 MB in length can be accepted, allowing analysis of whole chromosomes or substantial genomic regions. To fine-tune the identification of CpX islands, search parameters such as window size, CX percentage, observed/expected CpX ratio, and island merge gap can be customized. Individual default parameter settings based on previous experimental works are as follows:

#### Minimal window size

Set by default to 500 bp to indicate the smallest possible CpX island.

#### Minimum CX percentage

This represents the minimum percentage of CX dinucleotides within a window, with a default value of 55%.

#### Minimal observed/Expected CpX ratio

Relative to the expected CpX dinucleotides required for island identification, this ratio is set at 65% by default.

#### Maximal island merge gap

Defines the maximum distance between the islands detected to be merged; the default value is 100 bp.

#### First nucleotide

The first nucleotide is always cytosine.

#### Second nucleotides

With a predetermined value of G, the second nucleotide can be selected from G, A, T, or C.

### Methodology of detection

A sliding-window approach is used to detect CpX islands. It involves analyzing genomic sequences to find regions that meet defined requirements for the classification of CpX islands (the recommended parameters are present). This technique evaluates the quantity and dispersion of dinucleotides based on cytosine by combining neighboring qualifying windows to create continuous islands of CpX. With parameters that can be changed to alter detection sensitivity and account for the structural diversity of CpX islands across genomes, this procedure is essential to locate larger islands.

### Detection of CpX islands in the *Drosophila* genome

The genome of *D. melanogaster* was downloaded from NCBI (Genome assembly Release 6 plus ISO1 MT, accession number: GCF_000001215.4) and analyzed using CpX Hunter with the following (default) parameters: -minimal window size = 500 bp; -minimal CX percentage = 0.55; -Minimal Observed/Expected CpX Ratio = 0.65, and -maximal island merge gap = 100 bp. Results for particular CpX islands were downloaded in .BedGraph format (Supplementary materials 1, *A*–*C*) and further processed using Microsoft Excel 2021 to compute basic descriptive statistics. Violin plots, Chromosomal maps, and statistical testing were done using the SRplot web server (49) (accessed from https://www.bioinformatics.com.cn/srplot, 9th December 2024). CpX shores were obtained as regions 2 kbp upstream and 2 kbp downstream for the subset of long CpX.

### Analysis of CpX overlays with ChIP-Atlas data in *Drosophila*

The web server ChIP-Atlas 3.0 (50) (accessed from https://chip-atlas.org/, 1st December 2024) was used for making an “Enrichment Analysis” with the following parameters: Threshold for Significance = 100; Random permutations = x100 (for making a control dataset for comparison). The genome of *D. melanogaster* (version dm6) was selected. All experiment types (“ChIP: Histone”, “ChIP: RNA polymerase”, “ChIP: TFs and others”, “ATAC-Seq”, “DNase-Seq”, and “Bisulfite-Seq”) were subsequently inspected, always comprising “All cell types”. CpG, CpA, and CpT islands in .bed format were provided as inputs. Results were exported and are available as Supplementary material 2, *A*–*F*.

### Analysis of CpX overlays with TE data in *Drosophila*

Bedgraph coordinates of all natural TEs in the *D. melanogaster* genome were downloaded from the Flybase database (32): https://flybase.org/(accessed on 20th February 2025). An original bedgraph file is enclosed as Supplementary Material 3. Overlaps of TE data with CpX islands were computed within MS Excel.

### Functional enrichment analysis using STRING

STRING webserver v. 12.0 (51) (accessed from https://string-db.org/, 15th December 2024) was used for constructing functional networks from provided proteins and for obtaining the most statistically enriched GO terms. Organism *D. melanogaster* was used, and other parameters were left as the default.

## Data availability

All data presented in this study are freely available in the main manuscript, within supplementary materials, and on the publicly available web pages. The source code of the algorithm and web server: https://git.pef.mendelu.cz/bioinformatics/

Remote access: https://github.com/patrikkaura/dna-analyser-ibp/tree/master/.github.

The API is freely accessible on this web page: https://bioinformatics.ibp.cz/swagger-ui/index.html.

And use also this repository: https://git.pef.mendelu.cz/bioinformatics/backend.

## Supporting information

This article contains supporting information.

## Conflict of interest

The authors declare that they have no conflicts of interest with the contents of this article.
